# Role of glucosyltransferase R in biofilm interactions between *Streptococcus oralis* and *Candida albicans*

**DOI:** 10.1038/s41396-020-0608-4

**Published:** 2020-02-10

**Authors:** João Gabriel Silva Souza, Martinna Bertolini, Angela Thompson, Jillian M. Mansfield, André Alex Grassmann, Kendra Maas, Melissa J. Caimano, Valentim Adelino Ricardo Barao, M. Margaret Vickerman, Anna Dongari-Bagtzoglou

**Affiliations:** 1 Department of Oral Health and Diagnostic Sciences, University of Connecticut School of Dental Medicine, Farmington, CT USA; 2 Department of Prosthodontics and Periodontology, Piracicaba Dental School, University of Campinas (UNICAMP), Piracicaba, São Paulo Brazil; 3 Department of Oral Biology, School of Dental Medicine, University at Buffalo, Buffalo, NY USA; 4 Departments of Medicine, Pediatrics and Molecular Biology and Biophysics, University of Connecticut Health Center, Farmington, CT USA; 5 Microbial Analysis, Resources, and Services Core, University of Connecticut, Storrs, CT USA

**Keywords:** Microbiology, Biofilms

## Abstract

Streptococcal glucosyltransferases (Gtf) synthesize α-glucan exopolymers which contribute to biofilm matrix. *Streptococcus oralis* interacts with the opportunistic pathogen *Candida albicans* to form hypervirulent biofilms. *S. oralis* 34 has a single *gtf* gene (*gtfR*). However, the role of *gtfR* in single and mixed species biofilms with *C. albicans* has never been examined. A *gtfR* deletion mutant, purified GtfR, and recombinant GtfR glucan-binding domain were tested in single and mixed biofilms on different substrata in vitro. A mouse oral infection model was also used. We found that in single species biofilms growing with sucrose on abiotic surfaces *S. oralis gtfR* increased biofilm matrix, but not bacterial biomass. In biofilms with *C. albicans*, *S. oralis* encoding *gtfR* showed increased bacterial biomass on all surfaces. *C. albicans* had a positive effect on α-glucan synthesis, and α-glucans increased *C. albicans* accretion on abiotic surfaces. In single and mixed infection of mice receiving sucrose *S. oralis gtfR* enhanced mucosal burdens. However, sucrose had a negative impact on *C. albicans* burdens and reduced *S. oralis* burdens in co-infected mice. Our data provide new insights on the GtfR-mediated interactions between the two organisms and the influence of biofilm substratum and the mucosal environment on these interactions.

## Introduction

Glucosyltransferases (Gtfs) are streptococcal extracellular or cell-associated exoenzymes that hydrolyze sucrose and synthesize glucan polymers from the resulting glucose [1, 2]. These extracellular polymers, produced in various proportions of α-1,6 and α-1,3-linkages [3], contribute to the matrix that forms the scaffold for the three-dimensional architecture of biofilms, increasing bacterial adhesion, co-aggregation, and tolerance against antimicrobials [4–8]. Growth in the biofilm state occurs in many bacterial infectious diseases, thus glucans synthesized by Gtfs have been implicated as virulence factors [8].

Several oral streptococcal species have Gtf enzymes that may affect biofilm growth on abiotic, tooth, and mucosal surfaces. Although the Gtfs of *Streptococcus mutans* have been well characterized, due to their role in dental caries development, the biological roles of Gtf from the mitis group streptococci, including *S. oralis*, in oral biofilm communities are less clear. Streptococci of this group have been recognized as main initial colonizers in biofilms formed on tooth surfaces [9–11] and glucans have been implicated in facilitating biofilm accumulation [12, 13]. Members of the mitis group have a single Gtf-encoding gene, often regulated by a co-transcribed positive regulator, Rgg [13, 14]. Mitis group streptococci have been termed “accessory pathogens” due to their ability to form multispecies biofilms and enhance the community virulence [15].

*Candida albicans*–streptococcal interactions play an important role during the course of oral opportunistic infections [16–19]. Co-infection with *S. oralis* and *C. albicans* in a mouse model increased bacterial biofilms and severity of mucosal lesions, enhanced fungal pathogenicity, and resulted in an exaggerated inflammatory response [18]. *S. oralis* has a single Gtf structural gene (*gtfR*) [13]. GtfR has a high homology to other Gtf enzymes from mitis group streptococci, being able to synthesize water soluble and insoluble glucans using sucrose as substrate [13]. However, the role of GtfR and its α-glucan products in biofilm phenotypes has not been tested experimentally. We hypothesized that α-glucans synthesized by GtfR increase *S. oralis* biomass in biofilms growing with sucrose. We also hypothesized that GtfR-derived α-glucans modulate cross-kingdom interactions between *S. oralis* and *C. albicans* that lead to mutualistic relationships in mixed biofilms. In addition, because the type of substratum can affect microorganism adhesion and biofilm phenotype [20], we tested whether GtfR-mediated cross-kingdom interactions could be modulated by the type of biofilm substratum or by the mucosal environment in vivo.

## Materials and methods

### Strains and growth conditions

Strain construction in the *S. oralis* 34 parental background was done using standard molecular cloning techniques with modifications for oral streptococcal species [21] as described in Supplemental Material. *S. oralis* strains were reactivated from glycerol stocks by overnight growth in brain–heart infusion (BHI) medium (Becton, Dickinson and Company, Sparks, MD, USA) supplemented with antibiotics (Spectinomycin, 250 μg/ml, Erythromycin, 5 μg/ml) as needed, under static conditions at 37 °C, in a 5% CO_2_ incubator. *C. albicans* strain SC5314 was used as it forms robust biofilms with *S. oralis* 34 on abiotic and mucosal substrata and was grown as detailed previously [19].

### Single and mixed biofilm growth on abiotic surfaces

Biofilms of streptococci and *C. albicans* were allowed to develop for 6–48 h in RPMI 1640 medium supplemented with 10% FBS and 10% BHI [22, 23]. In 48 h biofilms, fresh media were added after 24 h. In some experiments media were supplemented with 1–5% [wt/vol] sucrose or 1% glucose [24]. Biofilms were grown on polystyrene (12-well plates and chamber slides) or titanium disks placed within the wells of 12-well tissue culture plates (American Society for Testing and Materials grade 2). Where indicated, surfaces were coated with FBS or dextran (100 μg/ml), for 30–60 min at 37 °C. Adhesion to coated and uncoated abiotic surfaces was assessed 1 h post inoculation, after washing nonadherent cells.

For biofilm growth, overnight stationary-phase cultures of *S. oralis* were inoculated in fresh BHI broth, allowed to reach exponential growth, and adjusted to OD_600_ = 1, representing a final suspension of 10^7^ cells/ml. Overnight cultures of *C. albicans* were prepared in YPD broth. The final inoculum in each biofilm consisted of 10^5^ cells of *C. albicans* and 10^7^ cells of *S. oralis*. In some experiments WT and *∆gtfR* strain biofilms were allowed to form for 24 h. Subsequently biofilms were washed with PBS and fresh media containing an inoculum of *C. albicans* (10^5^ yeast cells) were added and incubated for up to 16 h. Biofilms were incubated under static conditions at 37 °C in a 5% CO_2_ incubator.

### Biofilm growth on oral mucosal surfaces in vitro and in vivo

To examine the role of α-glucans in mucosal biofilm growth an oral stratified nonkeratinizing mucosal analog was used, described in detail previously [25, 26]. Tissues were infected with *C. albicans* (10^6^ cells/tissue), *S. oralis* 34 strains (WT and *ΔgtfR* mutant, 10^7^ cells/tissue) or a combination, in media supplemented with 1% sucrose [19]. Single and mixed biofilm growth was tested in vivo using a previously published mouse oral infection model, as described in Supplementary Methods [18].

### Microscopic analyses of biofilms

In some experiments Alexa Fluor 647-labeled dextran conjugate (1 µM; absorbance/fluorescence emission maxima, 647/668 nm) was added during biofilm growth, which is incorporated into α-glucans during matrix formation [27]. Biofilms were fixed with 4% paraformaldehyde for 2 h at 4 °C. *C. albicans* was visualized after staining for 2 h at room temperature using an FITC-labeled anti-*Candida* polyclonal antibody (Meridian Life Science, ME, USA). For biofilms containing streptococci, this was followed by fluorescence in situ hybridization with the EUB338 probe labeled with Alexa 405 or with Alexa 633-labeled probe [28, 29]. Biofilms were visualized by confocal microscopy. Stacks of *z*-plane images from at least three different fields of view per sample were reconstructed into 3-D images using IMARIS (Bitplane, Inc., Saint Paul, MN, USA). Surface reconstructions were used to calculate biovolumes and thickness. To visualize biofilms on mucosal constructs, tissue sections were stained as described above, and counterstained with Hoechst 33258 (Invitrogen, Carlsbad, CA, USA) to visualize epithelial nuclei. Biofilms growing on the tongue surface of infected mice were examined by scanning electron microscopy as described in Supplementary Material.

### Fungal and bacterial viable counts

Biofilms were vigorously vortexed in 2 ml PBS for 10 s, followed by sonication at 7 W for 30 s to break up cell aggregates. Mucosal tissues were homogenized followed by gentle sonication. Sonicates were serially diluted in PBS and 40 μl aliquots were plated on BHI agar supplemented with Nystatin (250 U/ml) for *S. oralis* 34 WT and on Sabouraud Dextrose Agar supplemented with chloramphenicol (1 mg/ml) for *C. albicans* quantification. For *ΔgtfR* strain plates were additionally supplemented with spectinomycin. *S. oralis* plates were incubated at 37 °C in an atmosphere of 5% CO_2_ and *Candida* plates at 30 °C in aerobic conditions for 2 days. Colony-forming units (CFUs) were counted by stereomicroscopy, and the results were expressed as log CFUs per biofilm.

### Gene expression analyses

Streptococcal RNA was purified from biofilms according to a published protocol which reduces the amount of extracellular polymers in biofilm matrix that interfere in RNA extraction [30]. The *gyrase* gene (*gyrA*) was used as internal control, which is stably expressed gene in *C. albicans*–streptococcal biofilms [31]. Data were calculated by the ∆∆Cq method and *gtfR* gene expression in co-species biofilms was expressed as fold relative to single species *S. oralis* biofilms [31].

*C. albicans* RNA from biofilms was extracted according to our previous protocol [22, 23]. For mucosal biofilms, tissues were homogenized using a POLYTRON® homogenizer and homogenates were beat with 0.5 mm zirconium beads (BeadBug® prefilled tubes, Sigma-Aldrich, St. Louis, MO, USA). *Efb1* gene was used as housekeeping control. Hyphae-associated genes *als3*, *hwp1*, and *efg1* were analyzed using primer sequences previously described [23].

### GtfR purification

GtfR secreted by wild-type *S. oralis* 34 was purified by chromatography according to a protocol [13] that results in active enzyme, as detailed in Supplementary Methods. Glucan synthesis was tested by coating polystyrene surfaces with purified enzyme (1 μg/ml in carbonate-bicarbonate buffer) for 1 h at 37 °C, followed by overnight incubation with Alexa Fluor 647-labeled dextran conjugate (1 µM), supplemented with 1% sucrose. A solution containing the fluorescently labeled probe but no sucrose served as negative control.

### Cloning and heterologous expression of recombinant GtfR glucan-binding domain

To assess the role of the glucan-binding domain (GBD) of GtfR in biofilms we generated a recombinant GBD (rGBD) in an *Escherichia coli* heterologous expression system. The 3-prime end of *gtfR* (encoding amino acids 1083 to 1554 of the 1575-residue GtfR protein) was amplified by PCR with primers NdeSo34GBDF and BamEngstopSo34GBDR2 (Table S1) and cloned in-frame with the compatibly digested *E. coli* expression vector pET28a for expression of rGBD with an N-terminal 6 × His tag. Expression and purification of rGBD are described in Supplementary Methods.

### Evaluation of *C. albicans* interactions with purified native GtfR and rGBD

To test the ability of native GtfR to modify *Candida* adhesion in a sucrose-independent manner, polystyrene wells were coated with purified protein as above and *C. albicans* (10^5^ yeast cells/well, suspended in RPMI, 10% FBS, 10% BHI) was added for 1 h. Surface area of adherent cells, stained with an FITC-labeled polyclonal antibody, was quantified microscopically using Image J. To test the sucrose-dependent GtfR effect on *Candida* adhesion 1% sucrose was added after coating, and glucans were allowed to form for 1 h at 37 °C. Wells were subsequently washed to remove residual sucrose, *C. albicans* was added and adhesion was assessed as above. Noncoated surfaces were used as control.

To test the ability of purified GtfR to adsorb to a preformed *C. albicans* biofilm and synthesize α-glucans, biofilms grown for 24 h on polystyrene wells were incubated for 1 h with 10 μg/ml of purified protein in carbonate-bicarbonate buffer [32]. After washing the unbound protein, glucans synthesized in situ were labeled by adding a solution containing Alexa Fluor 647-labeled dextran conjugate (1 µM) supplemented with 1% sucrose, incubated overnight, and examined by confocal microscopy.

To test the binding activity of *rGBD to C. albicans*, overnight YPD broth cultures (10^7^ yeast cells) were centrifuged and resuspended in PBS containing increasing concentrations of rGBD for 1 h at 37 °C. Cells were stained with 1:500 dilution of anti-6 × His-tag antibody conjugated to FITC (Biomatik®, Wilmington, DE, USA) and visualized by fluorescence microscopy and flow cytometry. rGBD binding to 24 h preformed *C. albicans* biofilms was tested similarly.

### 16S rRNA gene high-throughput sequencing

Tongue bacterial DNA and sequencing was performed using a lysis protocol and sequencing analysis pipeline optimized for mucosal microbiome characterization in murine tongue tissues [33]. The V4 region was amplified using 515F and 806R primers with Illumina adapters and bar codes on the 3′ end. Sequencing and data analysis details are listed in Supplementary Methods. All 16S V4 DNA sequencing raw data have been deposited in NCBI, SRA accession: PRJNA593873. Data are accessible at the following link: http://www.ncbi.nlm.nih.gov/bioproject/593873.

### Statistics

The Graph-Pad Prism software (Graphpad, La Jolla, CA, USA) was used for statistical analyses. Pair-wise and multiple group comparisons were done with the Bonferroni *t*-test and ANOVA, respectively, with significance set at *p* < 0.05.

## Results

### *S. oralis* glucosyltransferase affects biofilm structure on polystyrene surfaces

To test whether *gtfR* affects *S. oralis* biofilm formation and structure we first evaluated biofilm growth of *gtfR* mutant and complemented strains on polystyrene surfaces. To rule out intrinsic adhesion differences among these strains that could contribute to an altered biofilm phenotype, we allowed strains to adhere to either FBS–coated, dextran-coated, or uncoated polystyrene surfaces for 1 h, and then quantified attached organisms by CFU counts. We found no differences in adhesion between wild-type and mutant strains, although the complemented strain adhered more avidly to all surfaces (Fig. S1A).

**Fig. 1:**
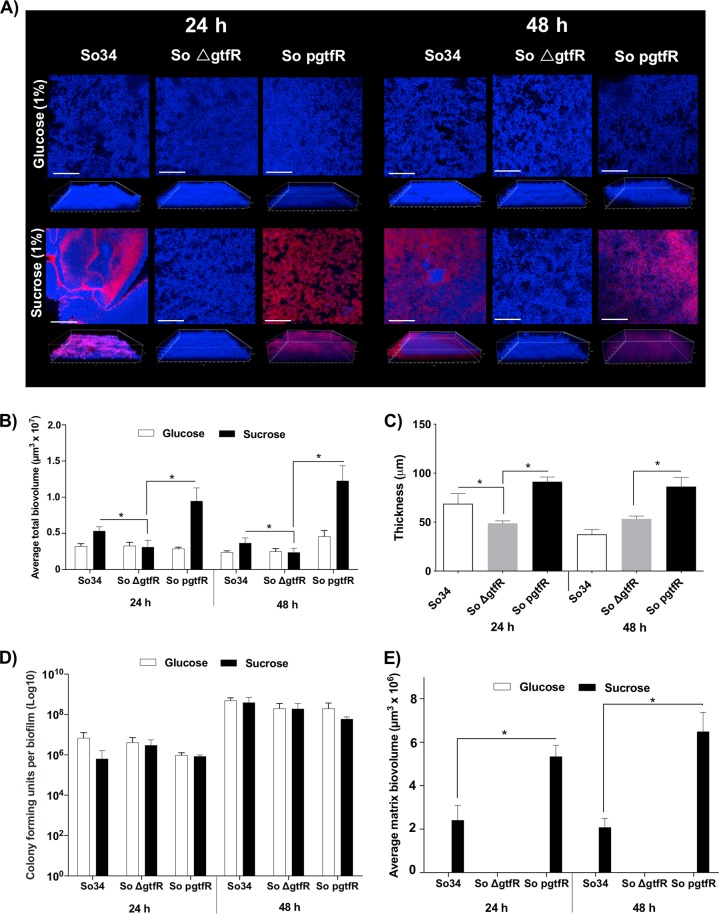
*S. oralis* biofilms growing for 24 or 48 h on polystyrene. Biofilms of wild-type (So34), *gtfR* mutant (So ∆gtfR) or complemented (So pgtfR) strains were grown in RPMI, 10%FBS, 10% BHI media supplemented with 1% sucrose or 1% glucose. **a** *X*–*Y* isosurfaces (top panel) and three-dimensional reconstructions (bottom panel) of representative confocal laser scanning microscopy images of biofilms. *S. oralis* (blue) was visualized after fluorescence in situ hybridization with a *Streptococcus*-specific probe conjugated to Alexa 405. Alexa Fluor 647-labeled dextran conjugate probe (red) was used to stain biofilm matrix (glucans). Scale bars, 50 µm (*X*–*Y* isosurfaces) and 70 µm (three-dimensional reconstructions). **b** Average total biovolumes (in µm^3^) for 24 and 48 h biofilms exposed to glucose (white bars) or sucrose (black bars) shown in (**a**) above. Biovolumes were measured in two different confocal laser scanning microscopy image stacks from two independent experiments. **c** Average biofilm thickness (in µm) in biofilms growing with 1%sucrose for 24 and 48 h. **d** Average *S. oralis* colony-forming units (CFU) in logarithmic scale for each 24 and 48 h biofilm. **e** Average matrix (α-glucan) biovolumes (in µm^3^) for 24 and 48 h biofilms. *p < 0.05, using the Bonferroni *t*-test. The error bars indicate standard deviations.

To assess α-glucan synthesis biofilm media were supplemented with sucrose as GtfR substrate, or glucose as negative control. Biofilms of parental and complemented strains growing in sucrose showed bacterial clusters enmeshed by α-glucan matrix in 24 h and 48 h biofilms (Fig. 1a). Under the same growth conditions Δ*gtfR* mutants were able to form biofilms, albeit devoid of α-glucans (Fig. 1a). Because the extracellular matrix is an important component of streptococcal biofilms [8], glucan production contributed to an increase in the total biofilm biovolume in both the wild type and complemented, as compared with the Δ*gtfR* strains (Fig. 1b). Parental and complemented strains formed biofilms with greater thickness (Fig. 1c, 24 h) and density, due to the presence of the extracellular matrix filling spaces between cell clusters (Fig. 1a). Differences in total biofilm biovolume in parental, *gtfR* deletion and complemented strains growing with sucrose could be explained solely by the observed differences in their glucan matrix biovolumes, because viable bacterial counts were not significantly different in these biofilms (Fig. 1d). A higher total biofilm (Fig. 1b) and matrix biovolume (Fig. 1e) were noted in the complemented compared with the wild-type strain, likely because it has multiple copies of plasmid-encoded *gtfR* and expresses higher levels of this gene (Fig. S1B).

### In biofilms with *C. albicans, S. oralis gtfR* promotes bacterial matrix and biomass

**Fig. 2:**
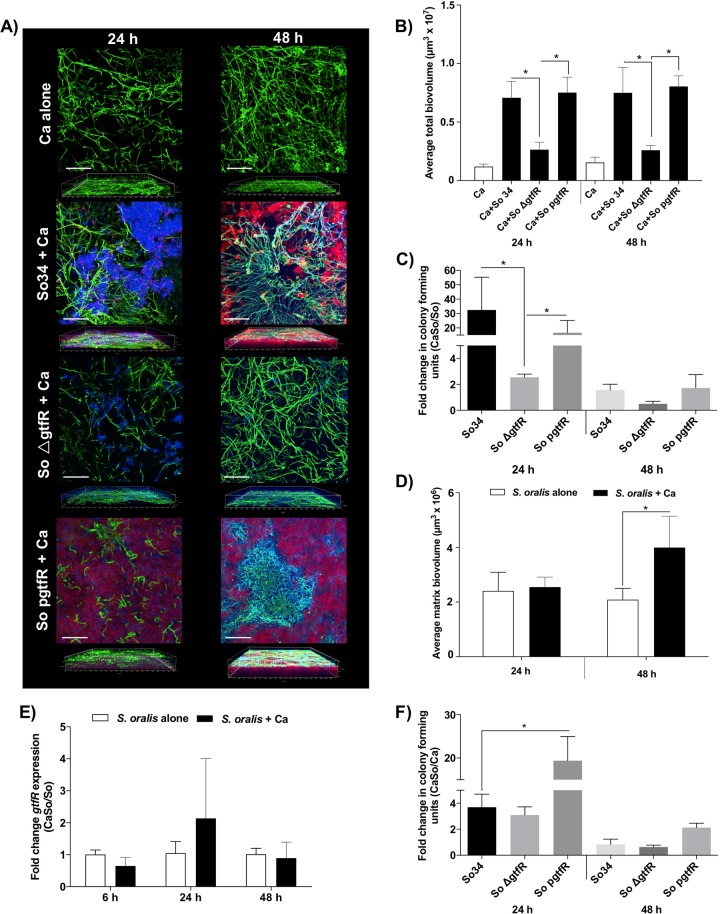
Twenty-four and forty-eight hour biofilms of *C. albicans* (Ca) alone or with wild-type *S. oralis* (So34), *gtfR* mutant (So ∆gtfR) or complemented (So pgtfR) strains. Biofilms were grown on polystyrene surfaces in RPMI, 10% FBS, 10% BHI media supplemented with 1% sucrose. **a** *X*–*Y* isosurfaces (top panel) and three-dimensional reconstructions (bottom panel) of representative confocal laser scanning microscopy images of biofilms. Please note overlap of green and red signals shown in yellow, suggesting close physical proximity between the two organisms. Scale bars 50 μm. **b** Average total biofilm biovolume (in µm^3^) for 24 and 48 h biofilms. **c** *S. oralis* CFU counts expressed as fold of mixed over single biofilms. **d** Average matrix biovolumes (in µm^3^). **e** Relative expression of *gtfR* gene levels assessed by RT-qPCR. Results represent mean fold change gene expression in *C. albicans* with *S. oralis* (CaSo, black bars) over *S. oralis* (So, white bars) biofilms alone, in three independent experiments. **f** *C. albicans* CFU counts expressed as fold of mixed biofilms with each of the three *S. oralis* strains (CaSo) over single biofilms (Ca). *p < 0.05, using the Bonferroni *t*-test. The error bars indicate standard deviations.

Growth of wild-type and complemented strains with *C. albicans* on polystyrene in 1% sucrose led to the development of dense biofilms enmeshed in α-glucan-rich matrix (Fig. 2a). Confocal images of mixed biofilms suggested physical proximity between α-glucan and *C. albicans* cells in 48 h biofilms (Fig. 2a). In contrast, mixed biofilms with the Δ*gtfR* mutant were sparsely distributed, although their thickness was similar to the wild-type strain (Fig. S1C). As expected, α-glucan synthesis by the WT and complemented strains contributed to a higher total mixed biofilm biovolume compared with the ∆*gtfR* strain (Fig. 2b).

In 24 h biofilms growing in 1% sucrose we found a significant, greater than twofold increase in *S. oralis* counts with all three strains in mixed biofilms compared with single *S. oralis* biofilms, suggesting that the positive effect of *C. albicans* on *S. oralis* biomass does not require GtfR activity. However, this effect was significantly higher in the wild-type and complemented strains compared with the ∆*gtfR* mutant, showing that even though *gtfR* is not required, it significantly enhanced this effect (Fig. 2c). In 48 h biofilms there was no significant effect of *C. albicans* on *S. oralis* viable counts for any strain, suggesting that differences in total biovolumes of late mixed biofilms among the three strains (Fig. 2b) are primarily due to differences in the amount of matrix produced. To further elucidate whether *C. albicans* has an effect on matrix production we compared single and mixed biofilms with the wild-type strain. The matrix biovolume in mixed biofilms with *C. albicans* was significantly higher compared with single *S. oralis* biofilms at 48 h, and although the average biovolume was higher at 48 h compared with 24 h this difference was not significant (Fig. 2d). The matrix increase in mixed over single biofilms at 48 h was not due to higher *gtfR* gene expression levels because expression was not different in mixed compared with single biofilms (Fig. 2e). These data suggest that on polystyrene surfaces *C. albicans* promotes an increase in streptococcal cell numbers in early stages of biofilm growth, leading to increased α-glucan-rich matrix synthesis in late stages. This work also shows the positive influence of *C. albicans* on *S. oralis* growth on polystyrene is primarily *gtfR*-dependent.

We next asked whether *gtfR* affects *C. albicans* growth in mixed biofilms. *S. oralis* strains conferred a modest growth advantage to *C. albicans* in 24 h biofilms, although this effect was more pronounced in biofilms with the complemented strain (Fig. 2f). We previously reported that in mixed mucosal biofilms *S. oralis* activates the *Efg1* filamentation pathway and increases hyphae-associated gene expression in *C. albicans* [23]. We thus tested whether *gtfR* influences these interactions by comparing the wild-type and mutant strains. Both the wild-type and Δ*gtfR* strains upregulated *Candida* genes in this pathway after 6 h of interaction however, there was no difference between the two strains (Fig. S2A).

### The type of substratum affects *gtfR*-associated biofilm phenotypes

**Fig. 3:**
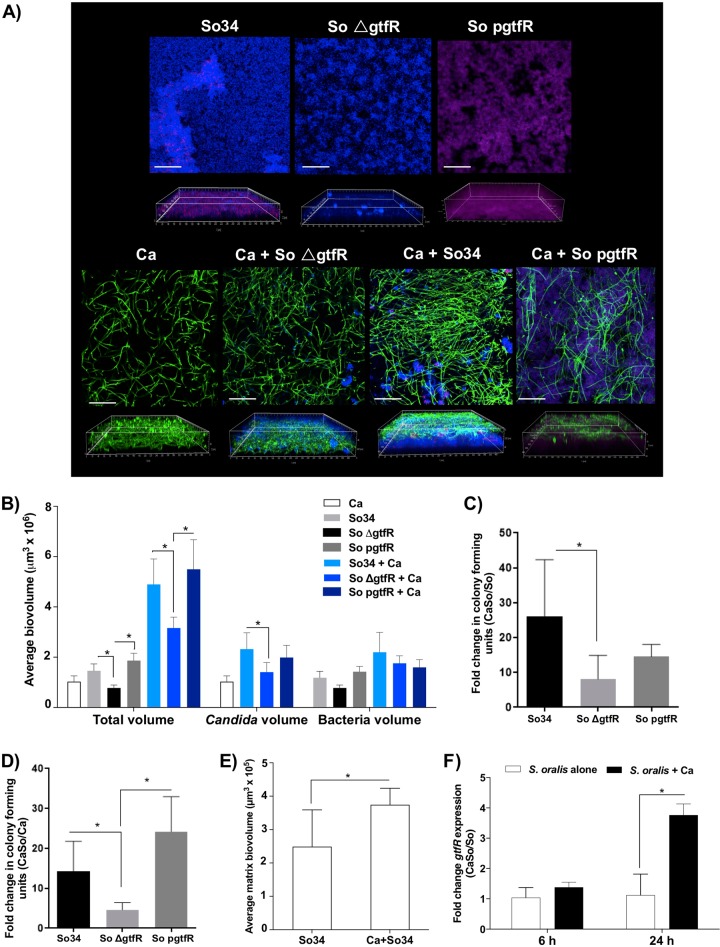
Biofilms growing on titanium surfaces for 24 h with 1% sucrose. *S. oralis* (So) (wild-type So34, ∆*gtfR* mutant, or complemented pgtfR strains) and *C. albicans* (Ca) growing alone or in combination. **a** *X*–*Y* isosurfaces (top panel) and three-dimensional reconstructions (bottom panel) of representative confocal laser scanning microscopy images of biofilms. Organisms and α-glucan-rich matrix were visualized by staining as above. Scale bars, 50 µm (*X*–*Y* isosurfaces) and 70 µm (three-dimensional reconstructions). **b** Average total, *Candida* or bacterial biovolumes (in µm^3^). Biovolumes were measured in two different confocal laser scanning microscopy image stacks from two independent experiments. **c** *S. oralis* CFU counts shown as mean fold of mixed biofilms over single biofilms in three experiments. **d** *Candida* CFU counts shown as fold of mixed biofilms over single biofilms. **e** Average matrix (α-glucans) biovolumes (in µm^3^) on *S. oralis* (WT) alone biofilms and *C. albicans*–streptococci mixed species biofilm. **f** Relative expression levels of *gtfR* gene in *S. oralis* strain 34 were analyzed by RT-qPCR. Results represent mean fold change gene expression in *C. albicans* with *S. oralis* (CaSo) over *S. oralis* (So) alone biofilms in independent experiments. *p < 0.05, using the Bonferroni *t*-test. The error bars indicate standard deviations.

Titanium is a commonly used biomaterial that supports mixed species biofilm growth [34]. We thus tested the *gtfR*-associated biofilm phenotype on this substratum. On titanium surfaces wild-type *S. oralis* formed thick biofilms enmeshed in α-glucan-rich matrix (Fig. 3a). Deletion of *gtfR* reduced the biofilm density and total biovolume, without a significant effect on bacterial biovolume (Fig. 3a, b) or CFU counts (data not shown) similar to the polystyrene phenotype. Confocal imaging showed sparsely distributed single biofilms of *C. albicans* on titanium surfaces (Fig. 3a).

On titanium we noted a robust mixed biofilm growth of the wild-type and complemented *S. oralis* strains with *C. albicans* (Fig. 3a), with significantly greater total biovolume compared with biofilms with the *gtfR* mutant (Fig. 3b). In biofilms with the wild-type and complemented strains we also noted a pronounced bacterial co-aggregation, with *C. albicans* mostly forming a layer over the streptococcal aggregates (Fig. 3a). There was a lower (albeit not statistically significant) bacterial biovolume and a significant reduction in bacterial viable counts between wild-type and mutant strains (Fig. 3b, c). In biofilms with wild-type *S. oralis* the fungal biovolume was significantly increased compared with single *C. albicans* biofilms (Fig. 3b). In contrast to polystyrene surface biofilms, fungal biomass on titanium was significantly higher in mixed biofilms with the wild-type *S. oralis* strain compared with the *gtfR* mutant, as reflected by biovolume and viable count estimates (Fig. 3b, d). These results suggest that in mixed biofilms GtfR promotes fungal accretion on titanium surfaces.

As seen in biofilms forming on polystyrene surfaces, mixed species biofilms of the wild-type strain with *C. albicans* had increased α-glucan matrix biovolume compared with monospecies biofilms (Fig. 3e). However, there were significantly lower amounts of matrix on titanium compared with polystyrene (Fig. S3). In 24 h mixed biofilms we also found an increase in *gtfR* gene expression compared with monospecies biofilms (Fig. 3f). In summary these data suggest that in mixed biofilms growing on titanium *gtfR* has a positive influence on both fungal and bacterial biomass. These results also show that on titanium surfaces *C. albicans* enhances both streptococcal cell numbers and *gtfR* expression, which could contribute to an increase in α-glucan-rich matrix synthesis in late stages.

**Fig. 4:**
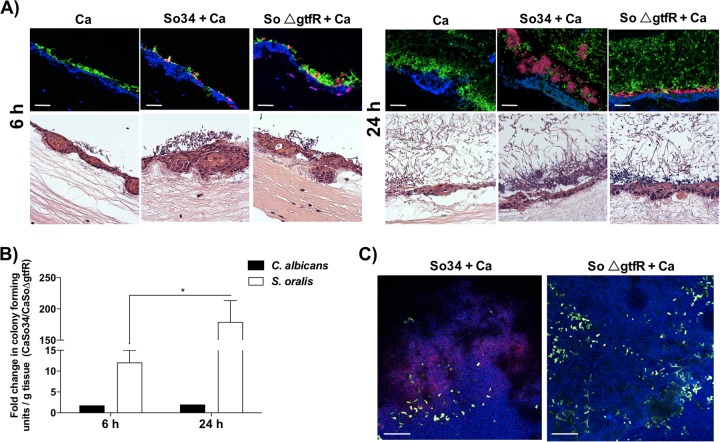
Biofilms of *C. albicans* alone or in combination with *S. oralis* (WT and ∆*gtfR* strains) growing on organotypic mucosal surfaces for 6 or 24 h. *C. albicans* (green) was visualized after staining with an FITC-conjugated anti-*Candida* antibody. *S. oralis* (red) was visualized after fluorescence in situ hybridization with a *Streptococcus*-specific probe conjugated to Alexa 546. **a** Tissue sections of mucosal biofilms with organisms stained as above, and mucosal cell nuclei counterstained with the nucleic acid stain Hoechst 33258 (blue, top panel). Corresponding haematoxylin and eosin-stained tissue sections are shown in the bottom panels. Scale bars 20 μm. **b** *C. albicans* (black bars) and *S. oralis* 34 (white bars) CFU counts expressed as fold of *Candida* and wild-type *S. oralis* mixed biofilms (CaSo34) over *Candida* and Δ*gtfR* mutant mixed biofilms (CaSoΔgtfR). **c** *X*–*Y* isosurfaces of representative confocal laser scanning microscopy images of mixed 24 h biofilms (green, *Candida*, blue, *S. oralis*) showing α-glucans (biofilm matrix) stained with Alexa Fluor 647-labeled dextran conjugate probe (red). Scale bars 50 μm. *p < 0.05, using the Bonferroni *t*-test. The error bars indicate standard deviation.

To extend these findings we next investigated the influence of *gtfR* in biofilms growing on an organotypic mucosa. Because *S. oralis* 34 wild-type strain alone does not form a biofilm on this surface [19] we focused on the influence of *gtfR* in mixed biofilms with *C. albicans*. Both Δ*gtfR* and wild-type strains formed a mixed biofilm with *C. albicans* on the mucosal surface (Fig. 4a). Similar to abiotic surfaces, there was a significantly greater bacterial biofilm growth in the wild type (and complemented, data not shown) compared with the Δ*gtfR* strain as assessed by viable counts (Fig. 4b), showing that *gtfR* promotes *S. oralis* biofilm growth with *C. albicans*. However, α-glucan matrix staining and biovolume estimates showed lower amounts of matrix produced by the wild-type strain in mixed biofilms on mucosa compared with other surfaces (Figs. 4c and S3).

Unlike biofilms growing on titanium, the fungal biomass on mucosal surfaces was not significantly affected by *gtfR* gene deletion, as shown by viable counts (Fig. 4b). Along the same lines *C. albicans* genes related to hyphal transformation were not influenced by *gtfR* gene deletion (Fig. S2B). Based on these findings we conclude that GtfR can enhance *C. albicans-S. oralis* mixed biofilm formation on mucosal surfaces by a positive effect on bacterial biomass, possibly by enhancing streptococcal cell–cell co-aggregation interactions, even though the α-glucan matrix was not a major component of these biofilms.

### GtfR-derived glucans promote *C. albicans* biofilm growth on abiotic surfaces

Prompted by the positive influence of *S. oralis gtfR* on *C. albicans* biofilm growth on abiotic surfaces, we further explored the mechanism of this interaction. *C. albicans* adhered poorly when inoculated in 1% sucrose-supplemented media on titanium surfaces, compared with polystyrene (Fig. S4A). We then hypothesized that α-glucans may promote adhesion of *C. albicans* which leads to greater biofilm growth. We first tested the effect of dextran, a soluble form of α-glucan, on adhesion and biofilm growth of *C. albicans*. Dextran coating improved adhesion of *C. albicans* to polystyrene (shown by reduction in nonadherent cells, Fig. S4B); however, this did not significantly affect *C. albicans* biofilm biomass as assessed by viable cell counts (Fig. S4C).

**Fig. 5:**
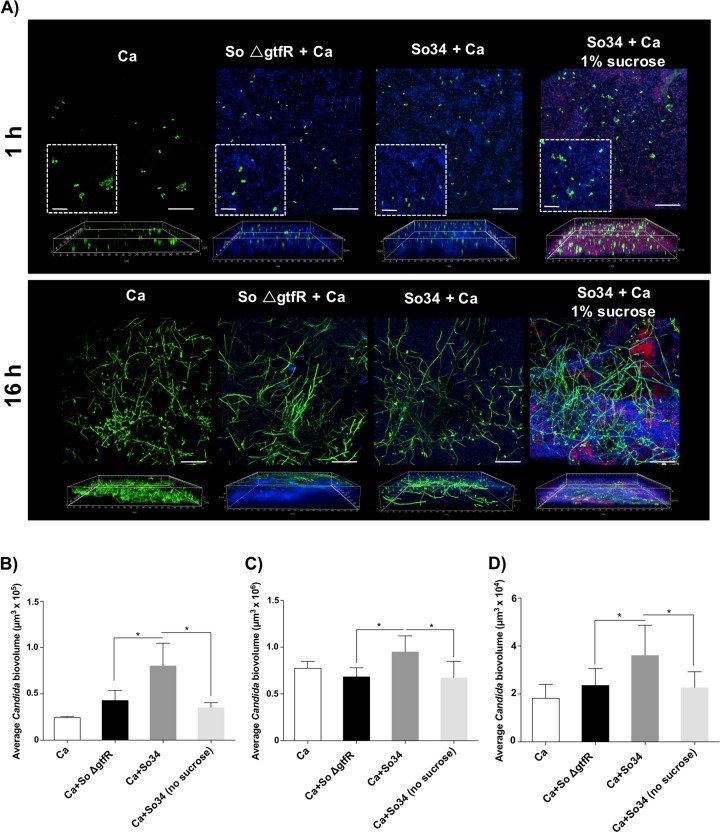
C. a*lbicans* interactions with preformed *S. oralis* biofilms. *S. oralis* biofilms were grown for 24 h using wild-type (So34) or ∆*gtfR* strains; media were supplemented with 1% sucrose or no carbohydrate. *C. albicans* was then added and incubated for 1 or 16 h. Unattached cells were washed and biofilms were stained. **a** *X*–*Y* isosurfaces (top panel) and three-dimensional reconstructions (bottom panel) of representative confocal laser scanning microscopy images of biofilms. *C. albicans* (green) was visualized after staining with an FITC-conjugated anti-*Candida* antibody. *S. oralis* (blue) was visualized after fluorescence in situ hybridization with a *Streptococcus*-specific probe conjugated to Alexa 405. Alexa Fluor 647-labeled dextran conjugate probe (red) was used to label biofilm matrix (α-glucans). Scale bars, 50 or 20 µm (*X*–*Y* isosurfaces) and 70 µm (three-dimensional reconstructions). Average *Candida* biovolumes (in µm^3^) after 1 h adhesion on *S. oralis* biofilms formed on polystyrene for 1 h (**b**) or 16 h (**c**). Similar experiments were performed on titanium surfaces and *C. albicans* biovolumes were quantified after 16 h (**d**). *p < 0.05 using the Bonferroni *t*-test. The error bars indicate standard deviations in triplicate experiments.

We then tested whether native α-glucans synthesized by wild-type *S. oralis* increase *C. albicans* biofilm accretion by a two-pronged approach. First we inoculated *C. albicans* yeast cells on preformed biofilms of the *ΔgtfR* or wild-type *S. oralis* strains (on polystyrene or titanium) and compared fungal biovolumes after 1 or 16 h of incubation. In the absence of sucrose, *C. albicans* biovolumes were not significantly different when fungi were inoculated directly on polystyrene or titanium, compared with inoculation onto a preformed Δ*gtfR* or WT strain biofilm (Fig. 5). However, a significant increase in fungal biovolumes was noted when *C. albicans* was inoculated on a preformed biofilm of wild-type *S. oralis* grown with sucrose and results were similar on both titanium and polystyrene surfaces (Fig. 5a–d). These results suggested that GtfR-synthesized α-glucans were responsible for increased *C. albicans* accretion.

**Fig. 6:**
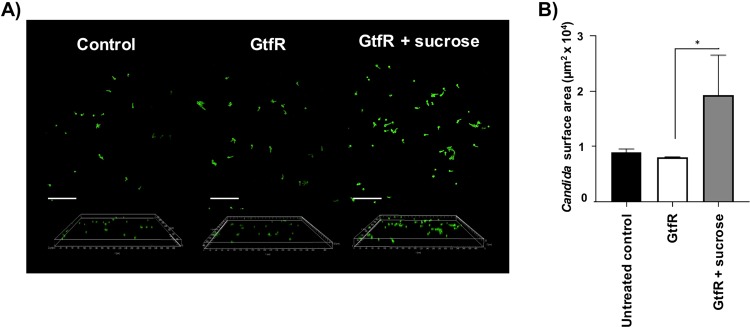
*C. albicans* adhesion on polystyrene surfaces. **a** Representative confocal images of *C. albicans*. Surfaces were precoated with purified GtfR (1 μg/ml) in the presence or absence of 1% sucrose for 1 h and *C. albicans* was inoculated after washing excess sucrose. *C. albicans* (green) was visualized 1 h post inoculation by staining with an FITC-conjugated anti-*Candida* antibody. **b** Average surface area covered by *Candida* cells as quantified by Image J analysis of three microscopic fields in each of two independent experiments. *p < 0.05 using the Bonferroni *t*-test. The error bars indicate standard deviations.

To strengthen these observations, in a second approach we explored whether α-glucans synthesized by the purified native enzyme from wild-type *S. oralis* 34 (Fig. S4D) promote fungal adhesion on abiotic surfaces. We first demonstrated that when polystyrene surfaces were coated with the purified enzyme, adding 1% sucrose led to synthesis of α-glucan which could be visualized coating the surface by confocal microscopy (Fig. S4E). When *C. albicans* was inoculated on α-glucan-coated surfaces there was significantly higher adhesion compared with untreated surfaces or surfaces that were coated with GtfR in the absence of sucrose (Fig. 6a, b). These results further suggest that α-glucan is responsible for the increase in *C. albicans* initial attachment and biofilm accretion on abiotic surfaces.

### GtfR binding to *C. albicans* does not play a role in interspecies interactions

Gtf proteins have a catalytic active site domain necessary for hydrolysis of sucrose, and a series of direct YG repeats in the carboxyl terminus thought to function in glucan binding. We next questioned whether the GBD of the GtfR protein could interact directly with *C. albicans* possibly leading to increased accretion or interspecies co-aggregation interactions in mixed biofilms. After optimizing the protein concentration needed to coat 96 well plates (Fig. S5A) we first tested the ability of rGBD-coated wells to bind biotin-conjugated dextran. All tested concentrations of dextran bound to the rGBD-coated wells (Fig. S5B). rGBD bound to the surface of only a small percentage of yeast cells (5–7%) as shown by immunofluorescence and FACS analyses (Fig. S6A). Increasing the concentrations of rGBD did not increase protein binding on *C. albicans* surface in the yeast form (Fig. S6B), or in early or late *C. albicans* biofilms (data not shown). We next examined whether purified native GtfR can bind to a preformed 24 h fungal biofilm and synthesize α-glucans on the surface of fungi after overnight incubation with 1% sucrose. We could not detect Alexa Fluor 647-labeled α-glucans on the surface of fungal cells (data not shown). We conclude that GtfR binding to fungal cells does not play a major role in the biofilm interactions of *C. albicans* and *S. oralis*.

### *S. oralis* mucosal biomass is enhanced by *gtfR* in vivo

**Fig. 7:**
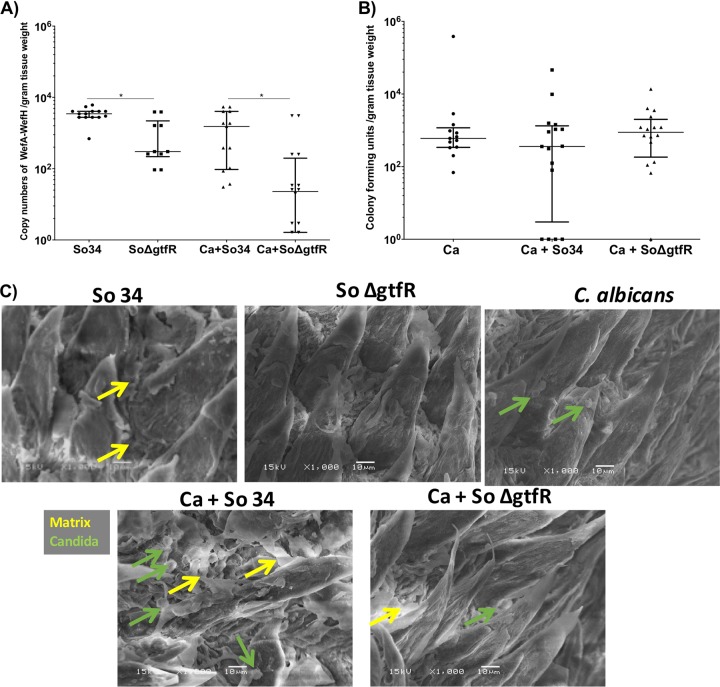
Role of *gtfR* in mucosal biofilms in vivo. Mice were inoculated with *S. oralis* wild-type (So34) or Δ*gtfR* strains, with or without *C. albicans* (Ca), and tongues were excised 5 days post inoculation at necropsy. **a** *S. oralis* mucosal burdens analyzed by qPCR using DNA extracted from tongues, and primers specific for the *S. oralis* 34 wefA-H gene. Cell (gene copy) numbers were calculated according to standard curves using known amounts of *S. oralis* 34 or Δ*gtfR* strain gDNA, and normalized over tissue weight. **b** *C. albicans* mucosal burdens as assessed by viable counts in tongue homogenates, normalized by tissue weight. Results of two independent mouse experiments, with 6–8 animals/group are shown. **c** Representative SEM images of biofilms forming on the tongue surface. Yellow arrows indicate the matrix-like material filling the spaces between filiform papillae in mice infected with wild-type *S. oralis* and *C. albicans*. Green arrows indicate yeast cells. *p < 0.05 using the Bonferroni *t*-test.

We next tested the role of *gtfR* in *S. oralis* mucosal biofilms in vivo. To unravel sucrose-dependent effects of GtfR mice received 5% sucrose in their drinking water [35]. In these experiments the wild-type mucosal burdens, as assessed by a strain-specific qPCR, were significantly higher than the Δ*gtfR* mutant in both single and mixed infections (Fig. 7a), and these results were confirmed by viable counts (data not shown). On the other hand the fungal biomass was not significantly affected by either the wild-type or mutant strains (Fig. 7b). In mice infected with *C. albicans* fungal cells were primarily in the yeast form, and in mixed infection with the wild-type *S. oralis*, yeast were interspersed within a matrix-like material occupying the space between the filiform papillae. This material was less abundant in mice infected with wild-type *S. oralis* only (Fig. 7c).

**Fig. 8:**
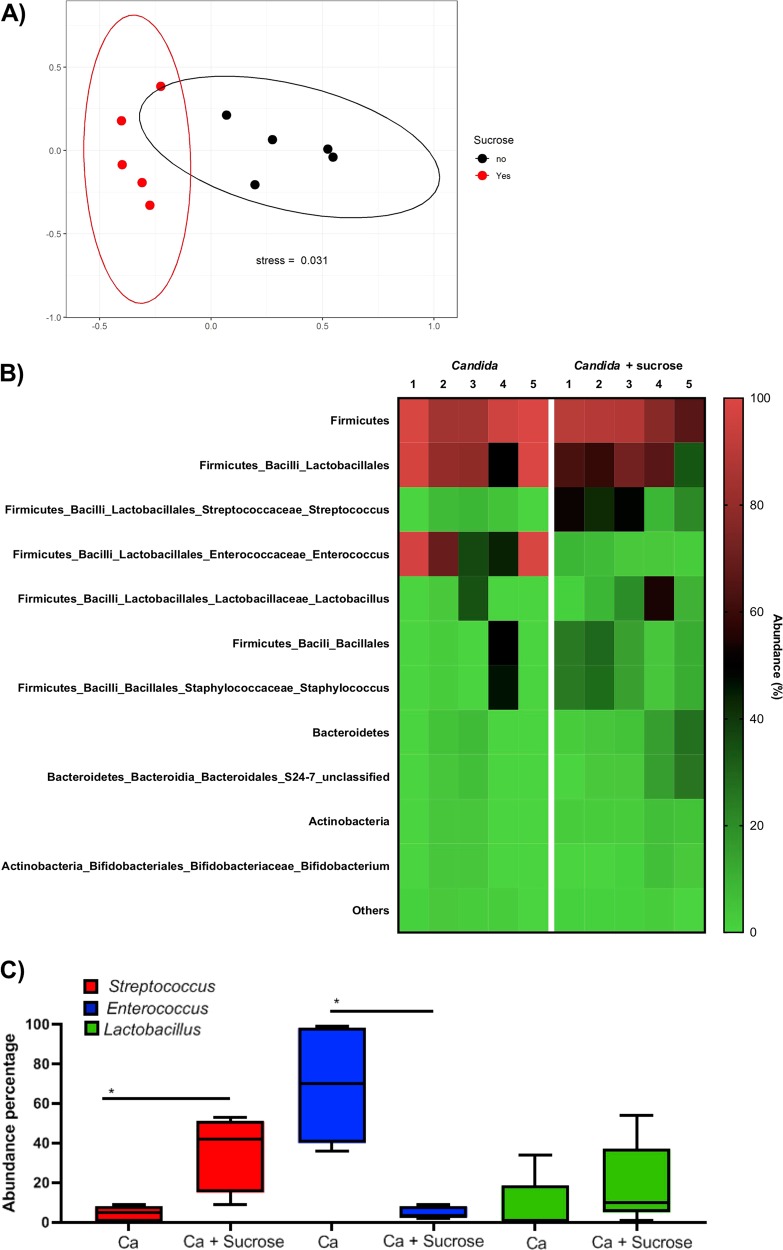
Mucosal bacterial microbiome analyses based on high-throughput 16S rRNA gene sequencing. **a** Beta diversity assessed by nonmetric multidimensional scaling (NMS) based on Bray–Curtis dissimilarities among the treatment groups. Shown are community structures in mice infected with *C. albicans*, in the presence or absence of added sucrose. Results represent bacterial community structure differences at the end of the experimental period (day 5). Communities clustered by type of treatment, indicating a significant effect of sucrose, which explained 52% of the variability (*p* < 0.02). **b** Relative abundance of bacterial 16S rRNA gene sequences corresponding to major mucosal genera in mice infected with *C. albicans*, in the presence or absence of added sucrose. **c** Relative abundance of endogenous *Streptococcus*, *Enterococcus*, and *Lactobacillus* in mice infected with *C. albicans* in the presence or absence of added sucrose, based on 16S rRNA gene sequences. *p < 0.05 for a *t*-test comparison of the two indicated groups.

Sucrose increased the mucosal burdens of wild-type *S. oralis* (Fig. S7A), but not the Δ*gtfR* mutant (data not shown). As published previously [18], *C. albicans* co-infection increased *S. oralis* burdens in mice not receiving sucrose. However, there was a significant drop in *S. oralis* burdens in co-infected mice receiving sucrose (Fig. S7A). Sucrose negatively influenced *C. albicans* burdens in both single and mixed infection models (Fig. S7B). These results suggested that the effect of sucrose on *S. oralis* burdens in co-infected mice was a consequence of the reduction in *Candida* burdens. We next hypothesized that the reduction in *Candida* burdens could be due to overgrowth of antagonistic bacteria promoted by sucrose. To test this hypothesis we assessed the impact of sucrose on the mucosal bacteriome of *Candida*-infected mice via 16S rRNA gene sequencing (Fig. 8). Nonmetric multidimensional scaling (NMS) analysis of Bray–Curtis dissimilarities showed that the bacterial microbiome composition was distinct in *Candida*-infected mice that received sucrose compared with ones that did not (Fig. 8a). Analysis of the most prevalent bacterial OTUs (minimum 1% of the reads in at least one sample/group) revealed distinct genus level differences between the two groups (Fig. 8b). We found that sucrose caused a statistically significant increase in the relative abundance of endogenous streptococci, whereas lactobacilli also increased but this did not reach statistical significance. On the other hand there was a statistically significant decrease in enterococci in *Candida*-infected mice receiving sucrose. (Fig. 8c).

## Discussion

Previous studies showed that *C. albicans* and *S. oralis* have a mutualistic relationship in the biofilm growth state which promotes fungal virulence [22, 23]. In this work we showed that *gtfR* promotes sessile growth of *S. oralis* on all tested surfaces in vitro and in vivo, when sucrose is available. However, in mixed biofilms with *C. albicans* the role of *gtfR* is modulated by the type of biofilm substratum and the mucosal environment. This may be due to the different amounts of α-glucan matrix on different substrata, which is more abundant on abiotic compared with mucosal surfaces. On abiotic surfaces *C. albicans* co-inoculation with *S. oralis* increased the amount of α-glucan matrix, by increasing bacterial cell numbers or *gtfR* gene expression. We showed that on these surfaces *C. albicans* adhesion and growth is promoted by the presence of preformed GtfR-synthesized α-glucans. Our studies are the first to provide experimental evidence that α-glucans synthesized by GtfR are important in cross-kingdom interactions between *C. albicans* and *S. oralis* favoring biofilm growth.

We found that the mucosal environment in vivo modulates sucrose-dependent interactions of the two organisms. One important finding was that sucrose reduces *C. albicans* mucosal burdens, in both single and mixed inoculation models. High glucose availability resulting from sucrose hydrolysis may repress *C. albicans* morphogenesis [36] and promote yeast dispersion [37]. This may explain the predominant yeast form on the tongue surface and the lower fungal burdens in mice receiving sucrose. Lower *C. albicans* burdens combined with reduced hyphae-associated cell wall proteins that enhance inter-kingdom interactions [17], may lead to reduction in *S. oralis* burdens in co-infected mice receiving sucrose. Exposure to a high sucrose diet changes the composition of the endogenous microbial communities in the alimentary tract of mice [38]. Such changes may involve overgrowth of endogenous lactic acid bacteria which curtail *C. albicans* growth and/or hyphal morphogenesis [39]. Consistent with this, our 16S rRNA gene analyses revealed an increase in abundance of lactic acid bacteria such as streptococci and lactobacilli in *Candida*-infected mice receiving sucrose. This is consistent with the positive effect of sucrose on exogenously supplied *S. oralis*. In contrast there was a reduction in enterococci in *Candida*-infected mice receiving sucrose. It is possible that, like lactobacilli, endogenous streptococcal species have antagonistic relationships with *C. albicans*. Of note, *S. oralis* and other human streptococcal species with symbiotic relationships with this fungus are not part of the mouse microbiome [18, 33, 40]. However, we have reported a symbiotic relationship between oral endogenous enterococci and *C. albicans* in mice [33], consistent with the reduction in the abundance of these organisms in mice receiving sucrose that have reduced *C. albicans* burdens.

The role of α-glucans has been mainly explored in relation to *S. mutans* and *C. albicans* and the development of dental caries [40]. *S. mutans* GtfB is able to bind avidly on mannans located on the outer surface *C. albicans* cell wall [41] and to synthesize extracellular α-glucans on the fungal surface [32]. In mixed biofilms forming on hydroxyapatite surfaces, *C. albicans* increases *S. mutans* extracellular biofilm matrix formation by upregulating *gtfB* and *gtfC* expression [24, 32]. In our studies *C. albicans* induced an upregulation of *gtfR* expression only on titanium surfaces, suggesting that similar to *gtfB* and *gtfC*, *gtfR* can be more actively transcribed when cells are adhering to this type of solid surface in the presence of sucrose [42]. The late (24 h) timing of this upregulation on titanium is consistent with the timing of *gtfB* and *gtfC* upregulation on hydroxyapatite surfaces [24]. Titanium surfaces were also associated with increased *C. albicans* biomass in mixed compared with single biofilms. It is possible that the physical and chemical properties of this biomaterial can affect protein adsorption on the surface modulating secondary fungal adhesion and biofilm formation [43–45]. Enhanced growth of *C. albicans* on titanium surfaces induced by pioneer dental plaque species such as *S. oralis* may promote mucosal inflammation and peri-implant disease and increased dietary sucrose exposure can amplify these effects [46].

Among the Gtf enzymes synthesized by *S. mutans*, only GtfB binds avidly to *C. albicans* and mediates co-aggregation interactions [32]. The selective binding of GtfB to *C. albicans* was suggested to be mediated by the carboxyl terminus of this protein, which contains the GBD and differs among the three Gtfs [32, 47]. Glucan-binding Gtf protein domains bind to α-1,6-linked glucosyl residues in glucans which confer a specific structural motif recognized as a binding site [48, 49]. GtfB binding to *C. albicans* is primarily mediated by mannans or mannoproteins exposed on the fungal cell wall [41]; however, the role of the GBD and how it affects selectivity of this Gtf in binding has never been examined. In our studies rGBD was not significantly adsorbed on the surface of *C. albicans*. This could be due to reduced *Candida*-binding function of the GtfR GBD or the requirement of a conformational structure for binding that is not attainable by producing the protein fragment in an *E. coli* host. Because there was also no detectable adsorption of the purified GtfR to *Candida*, direct binding may not play a major role in these interactions.

In conclusion, for the first time we showed that GtfR produces α-glucans which mediate cross-kingdom biofilm interactions between *C. albicans* and *S. oralis*. GtfR increased biofilm formation by a positive effect on bacterial biofilm matrix and biomass. This effect was modulated by the type of substratum and the mucosal environment in vivo.

## Supplementary information

Supplemental material

Supplemental figure 1

Supplemental figure 2

Supplemental figure 3

Supplemental figure 4

Supplemental figure 5

Supplemental figure 6

Supplemental figure 7
