# Supplementary material for: Role of glucosyltransferase R in biofilm interactions between Streptococcus oralis and Candida albicans
Source: ISME J. 2020 Feb 10;14(5):1207–22. doi: 10.1038/s41396-020-0608-4 (PMC7174356; doi:10.1038/s41396-020-0608-4)
Supplement: Supplementary file 1 — Supplemental material [file 41396_2020_608_MOESM1_ESM.doc]

**Supplemental material**

**Generation of isogenic *S. oralis* 34 *gtfR* deletion mutant and complement strains.**

Strain construction was done using standard molecular cloning techniques with modifications for oral streptococcal species [1]. Sequences of oligonucleotide primers used are shown in Table S1. Spectinomycin (250 µg/ml) and erythromycin (5 µg/ml) were used for transformant selection and plasmid maintenance as appropriate. To construct an isogenic mutant of strain 34 for which no nucleotide data were available, we first examined the genome for *rgg* and *gtfR* homologs. Primer sequences were inferred from sequences flanking *gtfR* and its upstream regulator *rgg* (loci SOR_RS06565 and SOR_RS06570, respectively) in the reference strain *S. oralis* Uo5 [2] (GenBank Accession NC015291). Primers Bam1349F and BamOralisdsR were used with strain 34 chromosomal template to amplify a 6.4-kb region encoding the putative polycistronic *rgg/gtfR* promoter and structural genes. The amplicon was digested with *Bam*H1 and ligated into the compatible site of a derivative [3] of the broad host range streptococcal replicative plasmid pVA749 encoding erythromycin resistance; the ligation mix was transformed into *S. gordonii* strain Challis CH1 as a cloning host. The resulting plasmid carrying *rgg* and *gtfR* was designated pGtfR. Strain 34 nucleotide sequences (GenBank Accession MK840882) for *rgg* and *gtfR* were determined to be 98% and 97% similar respectively to those of strain Uo5. Southern hybridization analysis of strain 34 chromosomal DNA probed with a *gtfR* internal fragment showed no additional *gtfR*-like DNA in strain 34 (data not shown).

To obtain DNA for construction of an isogenic mutant of *S. oralis 34* with an internal deletion of the *gtfR* gene, pGtfR was digested with *Eco*R1 and *Xho*I to release a 2.9-kb internal fragment of *gtfR*. A 1.2 kb *aad9* encoding spectinomycin resistance with compatible restriction sites was amplified by PCR and cloned into pGtfR. The 4.7-kbp dsDNA *Eco*R1/*Xho*I fragment, carrying *rgg* and *gtfR* with an internal deletion, was released from the vector by *Bam*H1 digestion and transformed with serum into the naturally competent *S. oralis* strain 34; putative transformants in which chromosomal allelic exchange had occurred were selected on spectinomycin agar. The appropriate chromosomal nucleotide sequence was confirmed by Southern hybridization analysis and nucleotide sequencing. Two biologically-independent *gtfR* deletion mutants were constructed. Both had similar soft colony phenotypes on sucrose agar plates indicating a lack of glucan synthesis and similar growth phenotypes. One strain was selected for further experimentation and designated *S. oralis* 34 *ΔgtfR*.

Genetic complementation of strain *S. oralis* 34 *ΔgtfR* was accomplished by transforming the strain with the multicopy plasmid pGtfR. Planktonic growth rates, determined turbidimetrically at OD_600_, were similar in the parental, mutant and complemented strains in all media used in this study (not shown).

**Cloning and heterologous expression of recombinant GtfR glucan binding domain**

To assess the role of the glucan binding domain (GBD) of GtfR in the growth of mixed species biofilms, we generated a recombinant GBD (rGBD) in an *E. coli* heterologous expression system. The 3-prime end of *gtfR* (encoding amino acids 1083 to 1554 of the 1575-residue GtfR protein) was amplified by PCR with primers NdeSo34GBDF and BamEngstopSo34GBDR2 (Table S1) and cloned in-frame with the compatibly digested *E. coli* expression vector pET28a for expression of rGBD with an N-terminal 6×His tag. The ligation mix was transformed into chemically competent *E. coli* DH5α for sequence verification and plasmid purification. The resulting pET28a/GBD was then transformed into *E. coli* BL21 Star™ (DE3) cells (Thermo Fisher), for expression in LB medium with kanamycin (50 µg/ml). When the culture reached mid-logarithmic phase (absorbance of 0.6-0.8 at OD_600_), at 37°C under agitation rGBD expression was induced by adding 1 mM Isopropyl β-D-1-thiogalactopyranoside (IPTG), followed by 4h incubation in same conditions. Cells were harvested by centrifugation, washed twice with PBS and lysed by adding lysis buffer (50 mM Na2HPO4, 500 mM NaCl, 1mg/ml Lysozyme, Xmg/ml DNAse, pH 7.5) and sonication. After centrifugation (12,000×g, 40 min, 4°C), the pellet was resuspended in Immobilized Metal Affinity Chromatography (IMAC) denaturing binding buffer (50 mM Na2HPO4, 500 mM NaCl, 20 mM Imidazole, 8M urea, pH 7.5) and centrifuged again (12,000×g, 40 min, 4°C). The supernatant was loaded in a HisTrap™ HP column (GE Health Care) for nickel-IMAC using the BioLogic DuoFlow™ automated system (BioRad). Aliquots containing purified recombinant protein were combined, dialysed against PBS, and checked by SDS-Polyacrylamide Gel Electrophoresis (SDS-PAGE) and Western blotting using anti-6X His tag® antibody conjugated to HRP (Abcam).

**Dextran binding activity of recombinant GBD**

We tested the *in vitro* dextran binding activity of rGBD as previously described [1]. Briefly, 0.5 μg/well of rGBD in coating buffer (0.05 M carbonate-bicarbonate, Sigma) was added to a 96-well plate and incubated overnight at 4°C. The plate was washed three times and blocked using 1% Bovine Serum Albumin (BSA) in PBS for 1h at room temperature. Different concentrations (15.6 ng to 2 μg/ml in PBS) of biotin-dextran (70,000 MW, Sigma) were added to the wells and incubated at room temperature for 2h. After three washes with PBS, 1:5,000 dilution of Streptactin:HRP (BioRad) was added, followed by 1h incubation at room temperature. After a final washing step, chromogenic substrate (TMB substrate solution, Thermo Fisher Scientific) was added, the reaction was incubated for 30 min at room temperature, stopped by adding 4N HPO4, and read at 405 nm. To confirm rGBD binding to the plate in preliminary experiments, a 96-well plate was coated with different rGBD concentrations (31 ng to 2 μg/well) in coating buffer, washed and blocked as described above, and 1:16,000 dilution of anti-6XHis tag® antibody conjugated to HRP (Abcam) was added followed by TMB substrate and read as described above.

**GtfR purification**

Overnight cultures were cultivated in 5 liters of dialyzed TTY media at 37°C (OD 0.8 at 550 nm), culture supernatants were collected by centrifugation and secreted protein was precipitated by saturation with ammonium sulfate. Then the precipitate was dissolved in 10 mM potassium phosphate buffer (pH 7.5) and dialyzed using the same buffer. Sample was loaded in a Hi Prep Q HP anion exchange chromatography column (GE Healthcare) and eluted by a linear gradient of 0 to 1.0 M NaCl. Purified protein was dialyzed using 10 mM potassium phosphate buffer (pH 6.0) and protein size (173 kD) was confirmed by SDS-PAGE.

**Mouse mucosal biofilm model**

Briefly, C57BL/6 mice (7-8 weeks old, Jackson Laboratory) were infected with *C. albicans* SC5314, *S. oralis* 34 wild type and Δ*gtfR* strains, or the combination of *C. albicans* and streptococci. On the first and third day of the infection protocol, mice were immunosuppressed by subcutaneous injection with cortisone acetate (225 mg/kg) dissolved in 200 ml PBS containing 0.5% Tween-20. On the second day, mice were anaesthetized by an intramuscular injection of ketamine:xylazine (90–100 mg/kg and 10 mg/kg of body weight, respectively) and a small cotton pad soaked with 100 µl of a *C. albicans* cell suspension (6x10^8^ yeast/ml), or 100 µl of streptococcal cell suspension (2.5x10^9^ bacteria/ml), or 50 µl of *C. albicans* cell suspension (1.2x10^9^ yeast/ml) combined with 50 µl of streptococcal cell suspension (5x10^9^ bacteria/ml), was used to swab the entire oral cavity. The swab was left for 2 h under the tongue and was removed before the animals awoke. During this time period animals were also given drinking water containing a daily-fresh suspension of *C. albicans* (6x10^8^ yeast organisms/ml) or streptococcal cell suspension (2.5 x10^9^ bacteria/ml) or the combination of the two. Water was supplemented with 5% sucrose, where indicated, as this has been shown to promote GtfR-mediated glucan synthesis by streptococci *in vivo* [5]. After sacrifice on day 5, tongues were excised and homogenized for bacterial and fungal CFU counts. Mitis-Salivarius® agar plates were used for *S. oralis* CFU counts on tongue tissues [6]. Tongue *S. oralis* burdens were also assessed by qPCR using a bacterial DNA extraction method and strain-specific primers detailed in our previous publications [6,7]. *S. oralis* 34-specific primer sequences span the adjoining sequence of two genes (*wefA-wefH,* *Forward: 5'- CATCAAGAACTTCTCGGAGTTG -3', Reverse: 5'-CCACAGCTCCAGAATATTTAGC-3'*) in the coaggregation receptor polysaccharide gene cluster [8]. A standard curve was set up with ten-fold serial dilutions of known amounts of gDNA from a pure culture of the wild type or mutant strains. Animal experiments were repeated twice with 5 mice per group. The study was approved by the University of Connecticut Health Center Animal Care Committee (Animal protocol #100358-0215).

**Scanning electron microscope (SEM) analysis**

To evaluate matrix production and tridimensional organization of biofilm on tongue surfaces, a scanning electron microscope (SEM) was used. Tongues were fixed in original Karnovsky’s mixture (5% Glutaraldehyde, 4% Formaldehyde in 0.064 M buffer) for 16 hours, followed by dehydration in a series of ethanol washes. Drying was achieved by a series of chemical washes with hexamethyldisilizane (HMDS), with a 1:2 solution of HMDS:100% ethanol. Samples were submerged in 100% HMDS in a fume hood overnight to dry aseptically. Tongues were mounted on stubs, sputter- coated with gold and examined with an SEM at 15 kV (model JSM5600LV, JEOL USA, Inc., Peabody, MA, USA).

**16S rRNA gene sequencing and analyses**

DNA was extracted from tongues using an overnight lysis protocol and the Qiagen DNA Blood and Tissue mini kit [7]. DNA was quantified using the Quant-iT PicoGreen kit (Invitrogen). 16S rRNA genes were amplified in triplicate using 30ng of extracted DNA as template. The V4 region was amplified using 515F and 806R primers with Illumina adapters and bar codes on the 3’ end [7]. PCR products were pooled for quantification and visualization using the QIAxcel DNA Fast Analysis kit (Qiagen). Pooled PCR products were processed using the Mag-Bind RxnPure Plus kit (Omega Bio-tek) according to the manufacturer’s protocol, to include only sequences between 250–400 bp. The cleaned pool was sequenced on the MiSeq using v2 2x250 base pair kit (Illumina).

Sequences were processed following a standard pipeline and classified using Mothur’s version of the Ribosomal Database Project classifier (Mothur 1.39.5) [9,10]. For operational taxonomic unit (OTU) analyses, sequences were clustered using a 97% similarity cutoff and classified up to genus level based on the consensus taxonomy. To assess the effect of sucrose on the mucosal bacterial microbiome the relative abundance of OTUs of the main genera was determined by subsampling 1000 reads per sample. Non-metric multidimensional scaling (NMS) plots were used to survey bacterial OTU heterogeneity relative to sucrose treatment and permutational ANOVA (PERMANOVA) comparisons of the Bray Curtis dissimilarity distances were performed. Community structures across groups were visualized in standard graphing packages within R, version 3.2 (http://www.r-project.org).

**References**

1. Vickerman MM, Sulavik MC, Minick PE, Clewell DB. Changes in the carboxyl-terminal repeat region affect extracellular activity and glucan products of *Streptococcus gordonii* glucosyltransferase. Infect Immun. 1996; 64:5117-5128.
2. Reichmann P, Nuhn M, Denapaite D, Brückner R, Henrich B, Maurer P, et al. Genome sequence of *Streptococcus oralis* strain Uo5. J Bact. 2011;193: 2888-2889.
3. Vickerman MM, Wang M, Baker LJ. An amino acid change near the carboxyl-terminus of the *Streptococcus gordonii* Rgg regulatory protein affects its abilities to bind DNA and influence expression of the glucosyltransferase gene, gtfG. Microbiology. 2003;149: 399-406.
4. LeBlanc, D. J., L. N. Lee, and J. M. Inamine. 1991. Cloning and nucleotide base sequence analysis of a spectinomycin adenyltransferase AAD(9) determinant from *Enterococcus faecalis*. Antimicrob. Agents Chemother. **35:** 1804–1810.
5. Kim D, Liu Y, Benhamou RI, Sanchez H, Simón-Soro Á, Li Y, et al. Bacterial-derived exopolysaccharides enhance antifungal drug tolerance in a cross-kingdom oral biofilm. ISME J. 2018;12(6):1427-1442.
6. Xu H, Sobue T, Thompson A, Xie Z, Poon K, Ricker A, et al. Streptococcal co-infection augments *Candida* pathogenicity by amplifying the mucosal inflammatory response. Cell Microbiol. 2014;16(2):214-31.
7. Bertolini M, Ranjan A, Thompson A, Diaz PI, Sobue T, Maas K, et al. *Candida albicans* induces mucosal bacterial dysbiosis that promotes invasive infection. PLoS Pathog. 2019;15(4):e1007717.
8. Yoshida Y, Ganguly S, Bush CA. Cisar JO. Molecular basis of L-rhamnose branch formation in streptococcal coaggregation receptor polysaccharides. J Bacteriol. 2006;188:4125–4130.
9. Kozich JJ, Westcott SL, Baxter NT, Highlander SK, Schloss PD. Development of a dual-index sequencing strategy and curation pipeline for analyzing amplicon sequence data on the MiSeq Illumina sequencing platform. Appl Environ Microbiol. 2013; 79(17):5112–20.
10. Wang Q, Garrity GM, Tiedje JM, Cole JR. Naive Bayesian classifier for rapid assignment of rRNA sequences into the new bacterial taxonomy. Appl Environ Microbiol. 2007;73(16):5261–7.

Table S1. Oligonucleotide primers used in PCR

| Primer name | 5’ to 3' sequence^1^ | Template |
| --- | --- | --- |
| Amplification of strain 34 *rgg/gtfR* region | | |
| Bam1349F | ATGGATCCGAGATGTCTTTGGAAATCAAC | *S. oralis* 34 chromosome |
| BamOralisdsR | ATGGATCCGCTCCTCTATGAATAATCAAGC | *S. oralis* 34 chromosome |
| Amplification of *aad9* to use in allelic replacement for mutant construction | | |
| EcoR1StopSpecF | ATGAATTC**TAA**TTTTCGTTCGTGAATACATG | pGem7:sp^R[4]^ |
| XhoSpecR | ATCTCGAGAAATCTGATTACCAATTAGAATG | pGem7:sp^R^ |
| Construction of pET28a:rGBD | | |
| NdeSo34GBDF | TACATATGACAACAGATGAGAAGATTACC | pGtfR |
| BamStopSo34GBDR2 | TAGGATCC**TTA**TTGAATCCATTTGCTTCGAGCC | pGtfR |

^1^ Engineered restriction sites for cloning are underlined. Engineered translational stops are in bold font.

**Supplementary Figure legends**

**Fig. S1 –** Adhesion of *S. oralis* on coated and uncoated polystyrene surfaces. (A) CFU of *S. oralis* wild type (So34), mutant (∆*gtfR),* and complemented strains (p*gtfR*) after 1 h adhesion in BHI supplemented with 1% sucrose. Polystyrene surfaces were uncoated or coated for 1 hour with FBS or dextran (100 μg/ml). (B) Relative expression levels of *gtfR* gene analyzed by RT-qPCR. Results represent mean fold gene expression in complemented over wild type strain in two independent cultures. (C) Thickness of 24 h mixed biofilms growing with 1% sucrose on polystyrene, based on confocal laser microscopic three-dimensional reconstructions. Results represent average thickness measured in two-three independent biofilms. **p*<0.05, using the Bonferroni t-test. The error bars indicate standard deviations.

**Fig. S2 –** Relative expression of *C. albicans* hypha-associated genes (*als3*, *hwp1*, *efg1*) in mixed biofilms with wild type *S. oralis* strain *34* or ∆*gtfR* mutant, expressed as fold over single *C. albicans* biofilms. Biofilms grew on polystyrene (A) or on oral mucosal analog surfaces (B) and were analyzed by RT-qPCR. Results from three independent experiments are shown.

**Fig. S3 –** Composite graph comparing total, bacteria, *Candida* and matrix biovolumes in mixed biofilms of *C. albicans* with wild type (So34) or ∆*gtfR* strains on polystyrene, titanium and mucosal organotypic surfaces. Different letters indicate statistically different surfaces for same mixed biofilm and biovolume measure (one-way ANOVA, *p*<0.05).

**Fig. S4 -** (A) *C. albicans* CFU counts representing adherent cells after 1 h inoculation on polystyrene or titanium surfaces, normalized by substratum surface area. (B) *C. albicans* CFU counts representing non-adherent (washed) cells after 1 h inoculation on uncoated or dextran (100 μg/ml)-coated polystyrene surfaces. (C) *C. albicans* CFU counts from cells that remained attached and formed biofilms for 20 h on the same surfaces as in (B). **p*<0.05. (D) SDS-gel of GtfR protein purified from wild type *S. oralis* 34. (E) α-glucans synthesized by purified GtfR. Polystyrene surfaces were coated with GtfR (1μg/mL) and then exposed or not to 1% sucrose for 1 h to confirm enzymatic activity of the purified protein. Synthesized glucans were labeled with an Alexa Fluor 647-labeled dextran conjugated probe, as above.

**Fig. S5 -** (A) Dose-dependent binding of recombinant GBD on 96 well polystyrene plates, as determined by ELISA using anti-6xHis:HRP antibody. X axis indicates rGBD protein concentrations in ng/ml. Results are expressed as absorbance at 450 nm. (B) Dextran binding of rGBD protein. Saturating amounts of rGBD (0.5 μg/well) were added to 96-well plates and incubated overnight. Increasing concentrations (0.0156 to 2 μg/ml in PBS) of biotin-dextran were then added and binding assessed as described in Supplementary Methods. Controls included Dextran (2μg) alone (i.e. no rGBD) or BSA (0.5 μg) added to Dextran. Results are expressed as absorbance at 405 nm.

**Fig. S6** - (A) Fluorescence images of planktonic yeast cells of *C. albicans* incubated with increasing concentrations of rGBD protein (0.5, 1, or 2 μg/mL) visualized with FITC-conjugated anti-6×His-tag antibody (green). (B) Quantification of rGBD protein binding to yeast cells by flow cytometry.

**Fig. S7 –**Effect of sucrose on tongue streptococcal and *Candida* burdens. Mice were inoculated with *S. oralis* wild type (So34) with or without *C. albicans* (Ca), and tongues were excised 5 days post-inoculation at necropsy. 5% sucrose was added to the drinking water daily in the groups receiving sucrose. (A) *S. oralis* mucosal burdens (B) *C. albicans* mucosal burdens. Fungal and bacterial burdens were analyzed by viable counts in tongue homogenates, and are expressed as log CFUs normalized by tissue weight. Results of two independent mouse experiments, with 6-8 animals/group are shown.
