# Supplementary figures and images for: Role of glucosyltransferase R in biofilm interactions between Streptococcus oralis and Candida albicans
Source: ISME J. 2020 Feb 10;14(5):1207–22. doi: 10.1038/s41396-020-0608-4 (PMC7174356; doi:10.1038/s41396-020-0608-4)

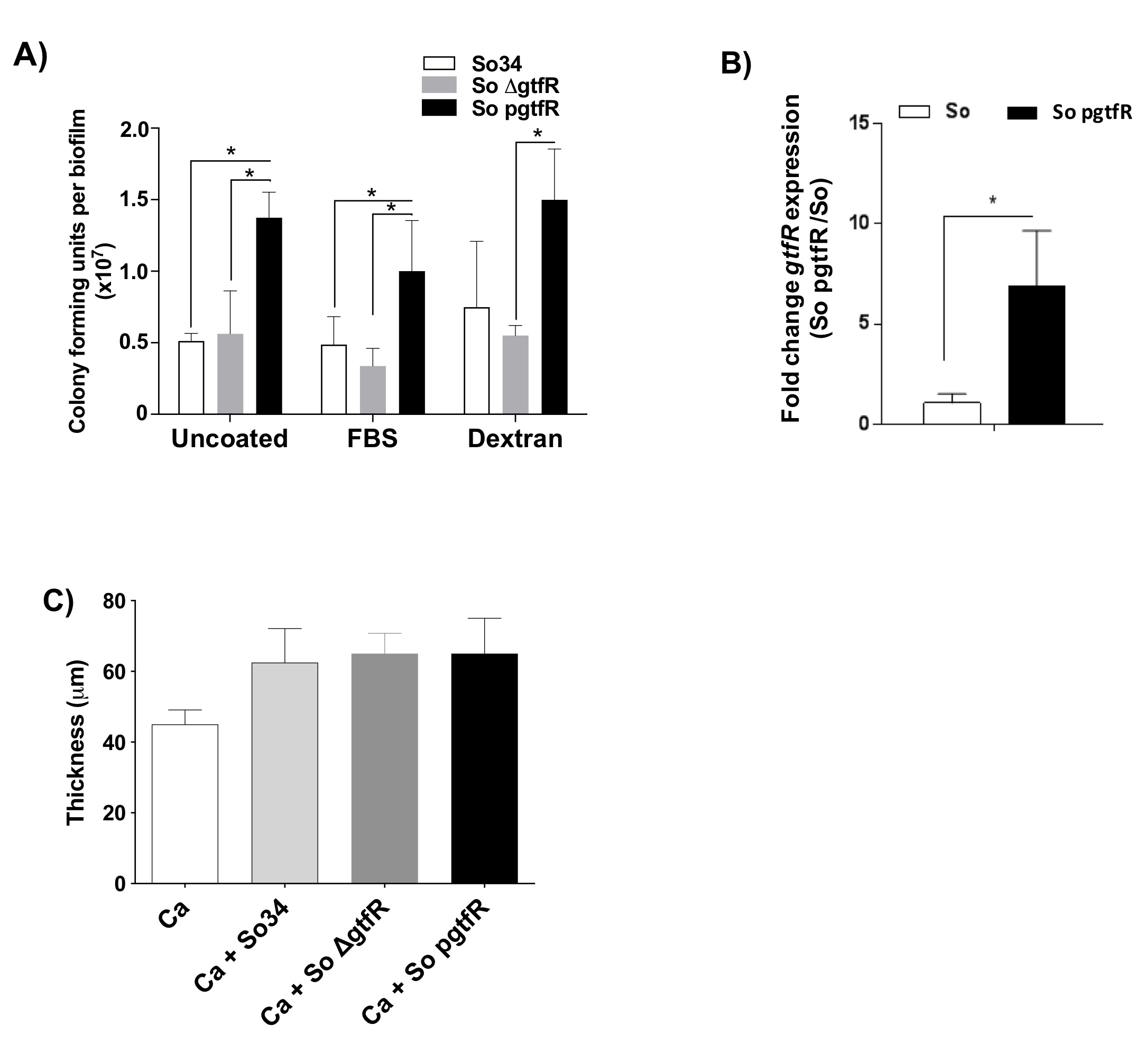

Supplement: Supplementary file 2 — Supplemental figure 1 [file 41396_2020_608_MOESM2_ESM.jpg]

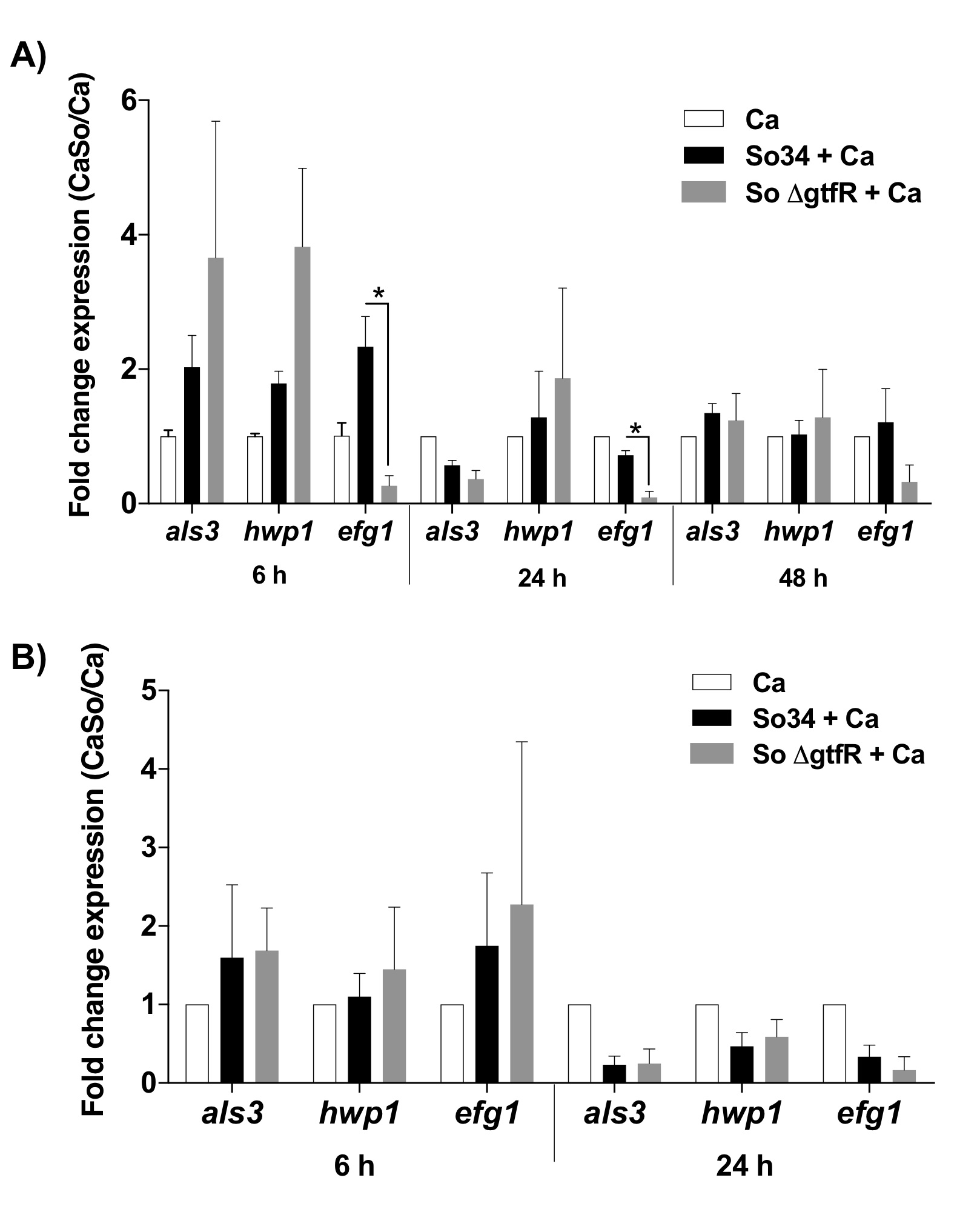

Supplement: Supplementary file 3 — Supplemental figure 2 [file 41396_2020_608_MOESM3_ESM.jpg]

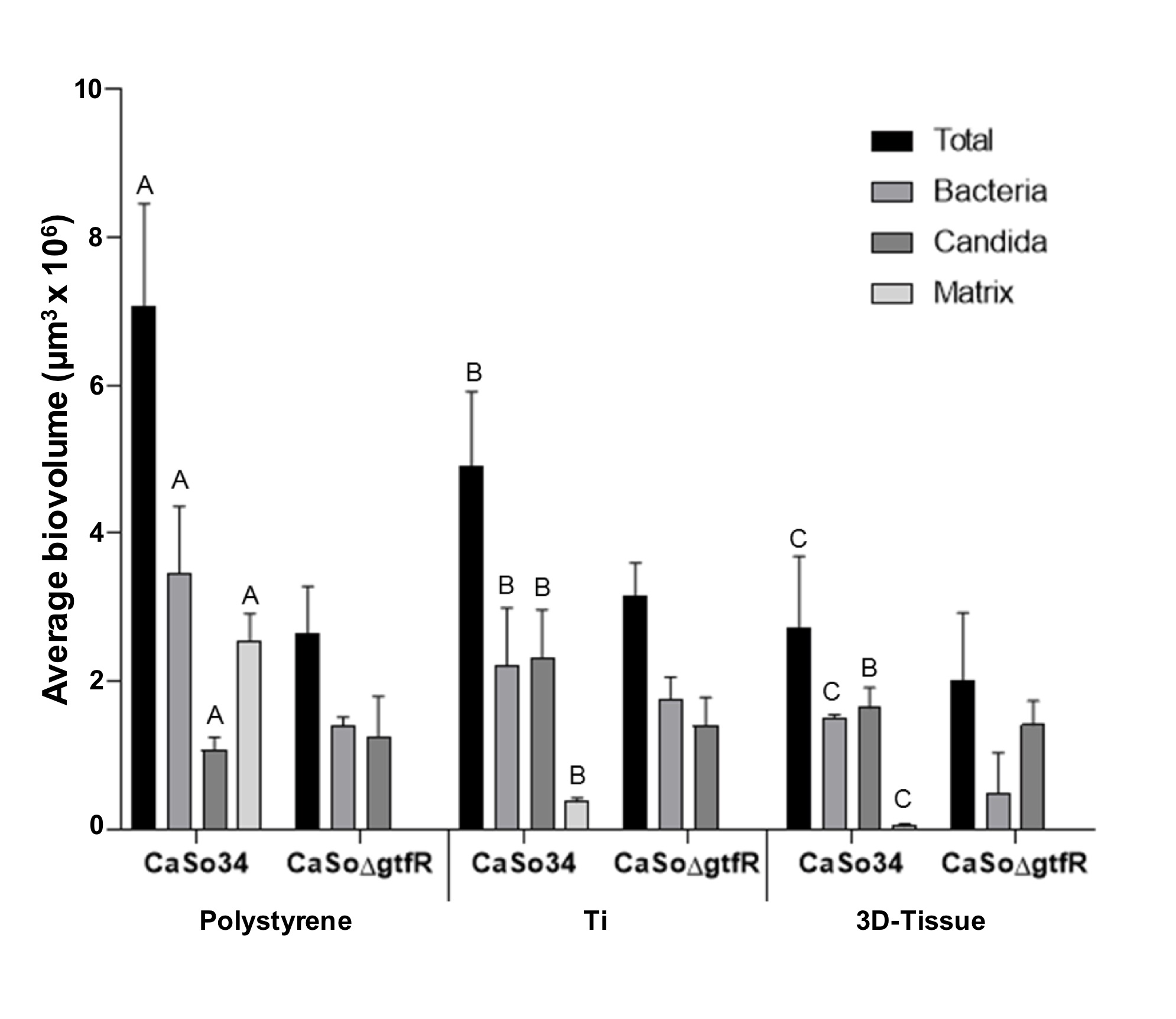

Supplement: Supplementary file 4 — Supplemental figure 3 [file 41396_2020_608_MOESM4_ESM.jpg]

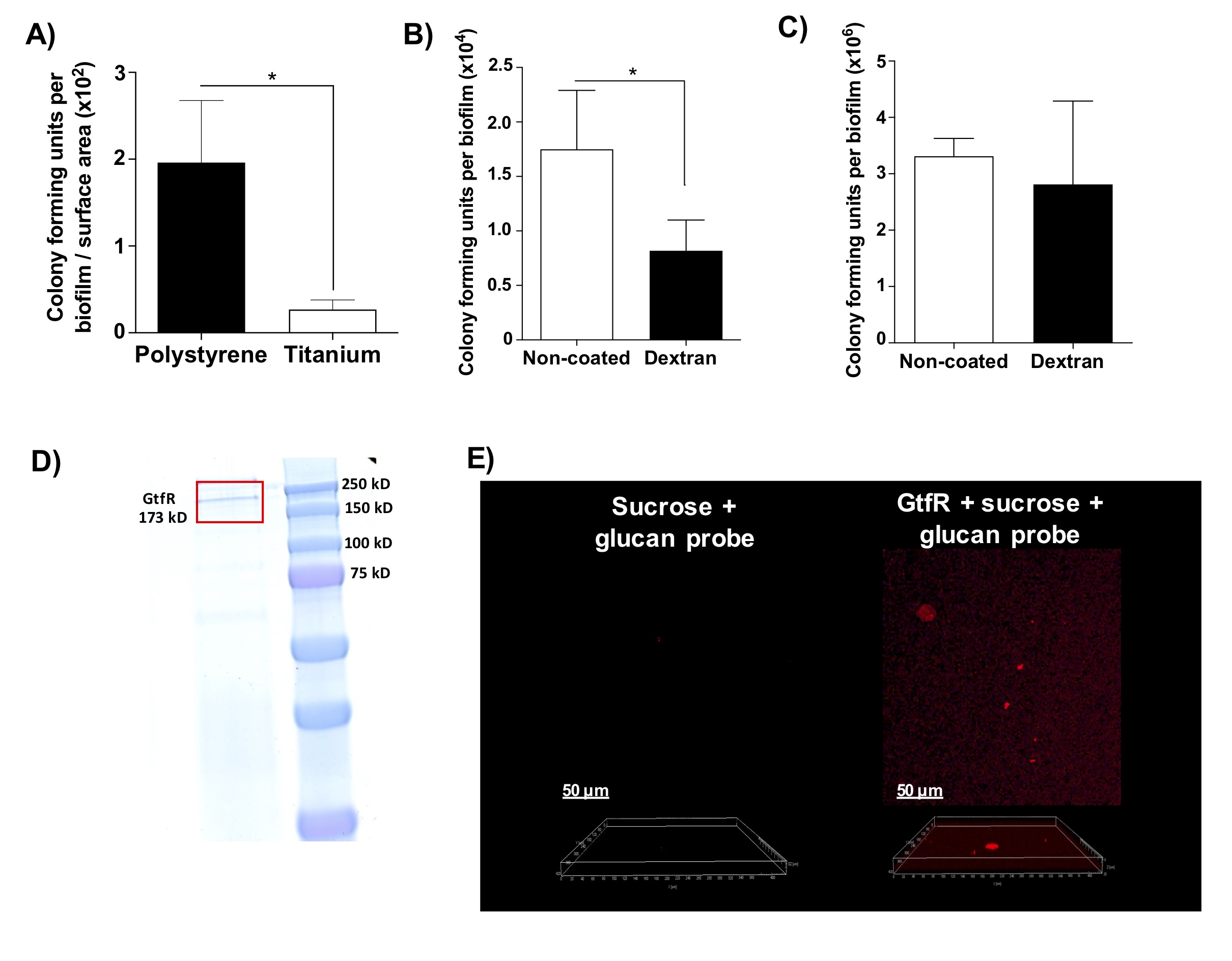

Supplement: Supplementary file 5 — Supplemental figure 4 [file 41396_2020_608_MOESM5_ESM.jpg]

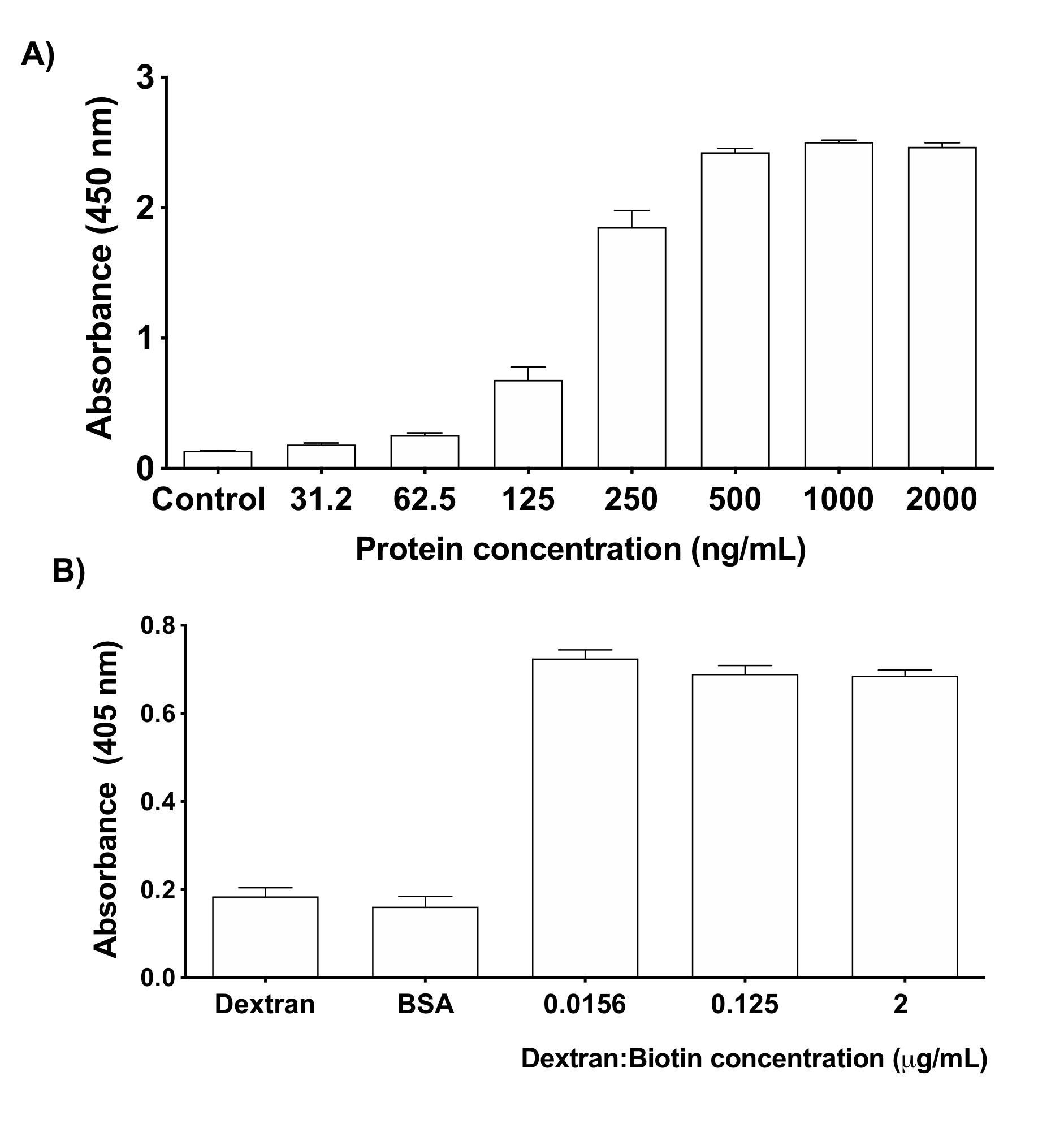

Supplement: Supplementary file 6 — Supplemental figure 5 [file 41396_2020_608_MOESM6_ESM.jpg]

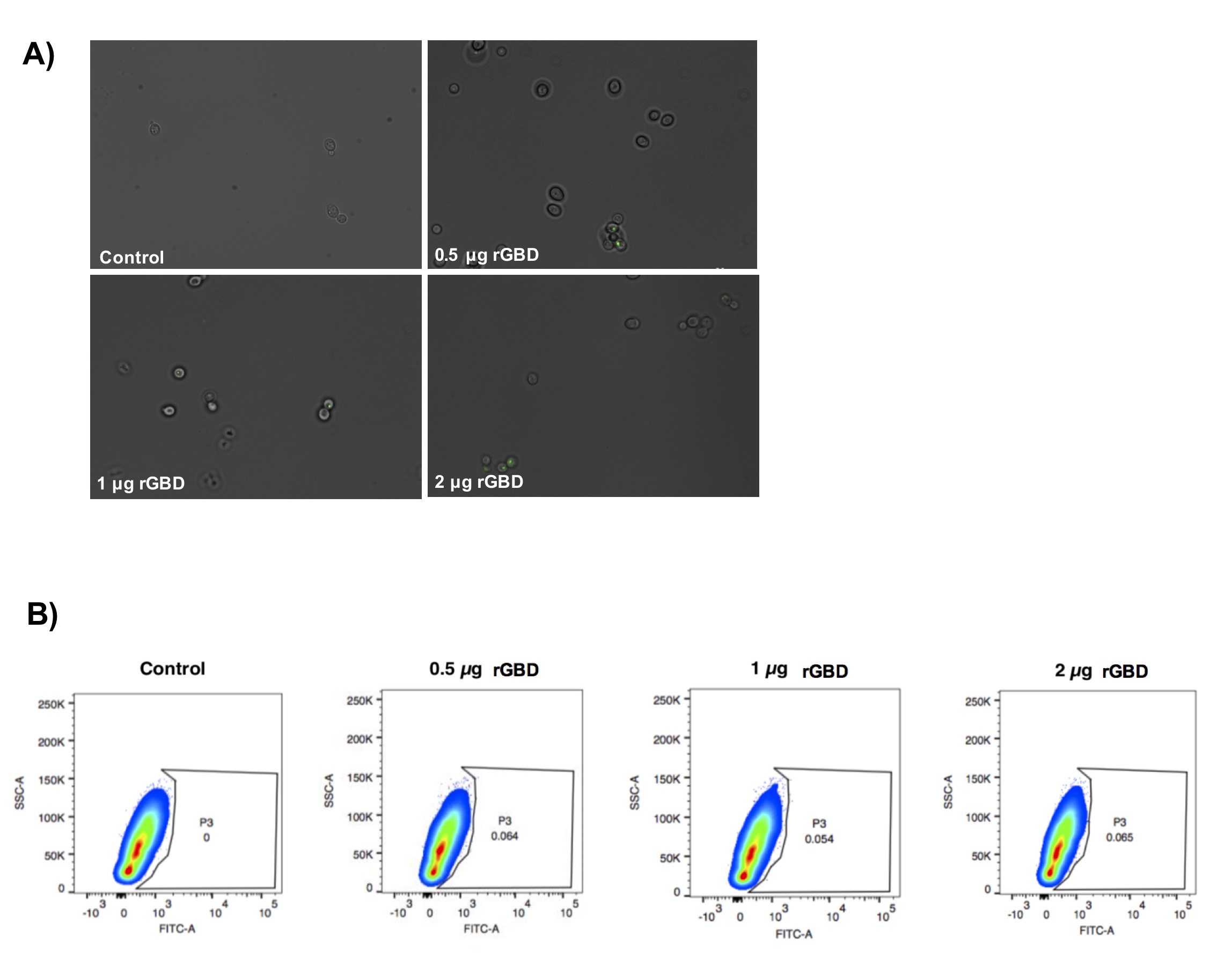

Supplement: Supplementary file 7 — Supplemental figure 6 [file 41396_2020_608_MOESM7_ESM.jpg]

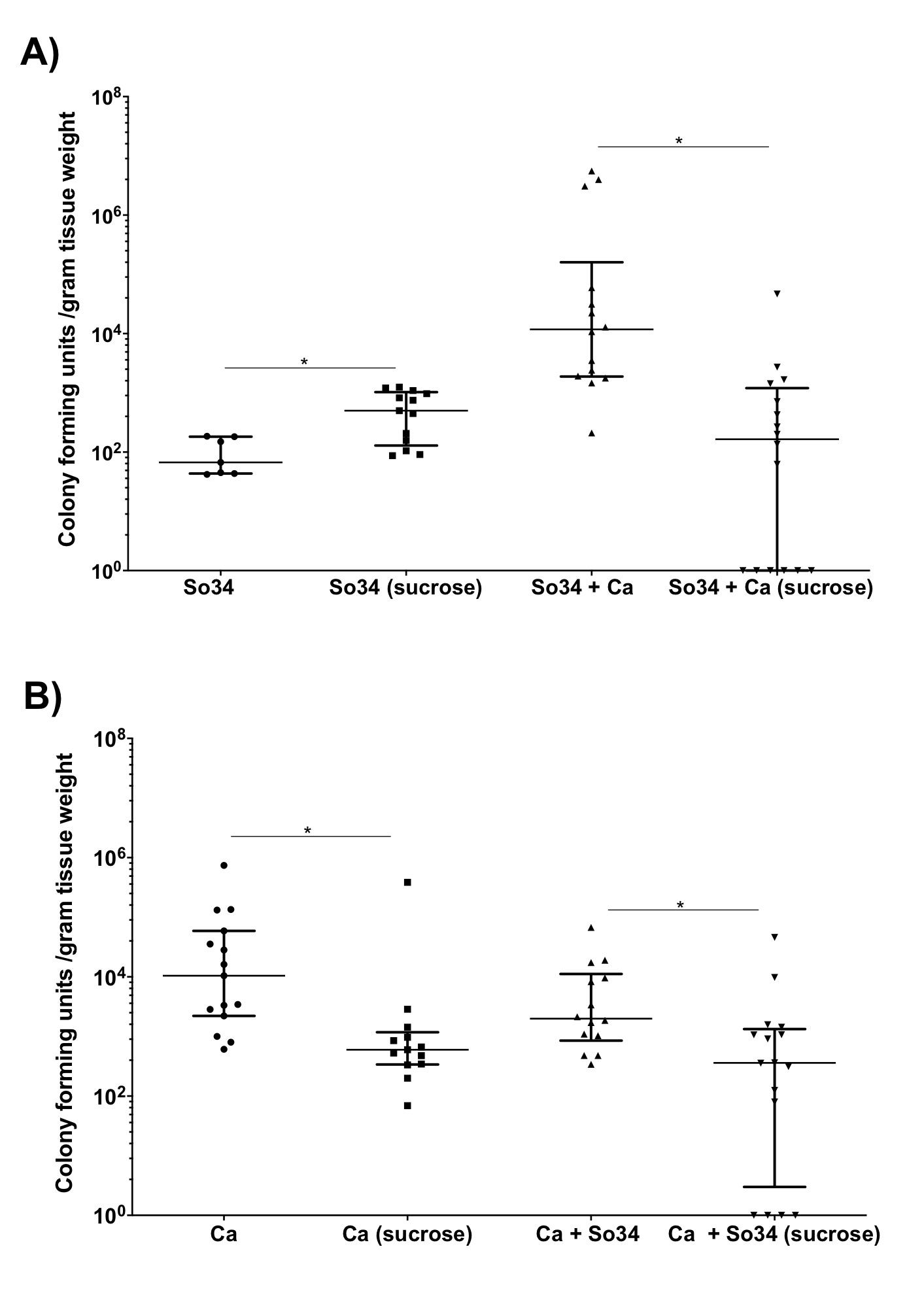

Supplement: Supplementary file 8 — Supplemental figure 7 [file 41396_2020_608_MOESM8_ESM.jpg]
